# Findings from a pilot open-label trial of N-acetylcysteine for the treatment of pediatric mania and hypomania

**DOI:** 10.1186/s12888-022-03943-x

**Published:** 2022-05-03

**Authors:** Janet Wozniak, Maura DiSalvo, Abigail Farrell, Carrie Vaudreuil, Mai Uchida, T. Atilla Ceranoglu, Gagan Joshi, Emmaline Cook, Stephen V. Faraone, Joseph Biederman

**Affiliations:** 1 Clinical and Research Program in Pediatric Psychopharmacology and Adult ADHD, Massachusetts General Hospital, 55 Fruit St., Warren 705, Boston, MA USA; 2 Department of Psychiatry, Harvard Medical School, Boston, MA USA; 3 Department of Psychiatry and Behavioral Sciences, SUNY Upstate Medical University, Syracuse, NY USA

**Keywords:** Bipolar spectrum disorder, Child, N-acetylcysteine

## Abstract

**Background:**

Pediatric bipolar disorder is a highly prevalent and morbid disorder and is considered a prevalent public health concern. Currently approved treatments often pose the risk of serious side effects. Therefore, this study assessed the efficacy and tolerability of N-acetylcysteine (NAC), in children and adolescents with bipolar spectrum disorder.

**Methods:**

We conducted a 12-week open-label trial of NAC for treatment of mania and hypomania in children and adolescents ages 5–17 with bipolar spectrum disorder including participants with full and subthreshold manic symptoms, accepting those with and without mixed states with co-occurring depression, and Young Mania Rating Scale scores ≥ 20 and < 40. Symptoms of mania and depression were assessed using the Young Mania Rating Scale (YMRS), Hamilton Depression Rating Scale (HDRS), Children’s Depression Rating Scale (CDRS), and Clinical Global Impression (CGI) Severity (CGI-S) and Improvement (CGI-I) scales for mania and depression.

**Results:**

This study had a high drop-out rate with only 53% completing all 12 weeks. There was a significant reduction in YMRS, HDRS, and CDRS mean scores from baseline to endpoint. Of the 24 exposed participants, 54% had an anti-manic response measured by a reduction in YMRS ≥ 30% and 46% had a CGI-I mania score ≤ 2 at endpoint. Additionally, 62% of participants had an anti-depressive response measured by a reduction in HDRS ≥ 30%, 31% had an anti-depressive response measured by a reduction in CDRS ≥ 30%, and 38% had a CGI-I depression score ≤ 2 at endpoint.

**Conclusions:**

These pilot open-label findings in a small sample provide preliminary data supporting the tolerability and safety of NAC in a pediatric population. The findings of this pilot scale study indicating improvement in mania and depression are promising, but require replication with a monotherapy randomized placebo controlled clinical trial and larger sample.

**Trial Registration:**

ClinicalTrials.gov Identifier: NCT02357290. First Registration 06/02/2015.

## Background

Due to its high prevalence of 1–3% in national and international samples and morbidity, pediatric bipolar (BP) disorder represents a serious public health concern [1–4]. Pediatric BP disorder is commonly characterized by high levels of severe irritability and concurrent features of both mania and depression, which may complicate the diagnosis and treatment of youth with this disorder. Children with BP disorder also have high rates of psychiatric comorbidity with attention-deficit/hyperactivity disorder (ADHD), anxiety disorders, conduct disorder, oppositional defiant disorder (ODD), substance use disorders, and disruptive behavior disorders; high rates of psychiatric hospitalization; and need for special education services [5–9]. Pediatric BP disorder is similarly morbid when present in subsyndromal or subthreshold forms [10, 11]. It is also a well-documented risk for suicide, a leading cause of mortality in the young [12, 13]. Additionally, because of the severity of their aggressive behavior, youth affected with BP disorder are often diverted to the juvenile criminal justice system [14–16].

Pediatric BP disorder is persistent. Three follow-up studies indicate that pediatric onset BP disorder persists or worsens over time, with subthreshold cases switching to full threshold status during follow-up periods and youth spending the majority of time in manic, depressed and mixed mood states. Geller et al. demonstrated that the frequency of manic episodes in grown-up subjects with pediatric BP-I was 13 to 44 times higher than population prevalences, strongly supporting continuity of childhood BP into adulthood [17]. In the Course and Outcome of Bipolar Youth (COBY) study 25% of BP-II and 38% of BP NOS pediatric participants converted to BP-I during the follow-up period, and subjects were symptomatic on average for 60% of the follow-up period [18]. When attending to types of remission, few pediatric subjects experience full syndromatic remission, and even fewer achieve functional remission [19].

While the pathophysiology of pediatric BP disorder remains elusive, emerging evidence points to potential neurolobiologic underpinnings, such as abnormalities of glutamate [20] and white matter abnormalities the cingulum bundle areas [21]. Familiality of pediatric BP has been firmly established in large family studies [22]. A genome wide association study found shared genetic risks between ADHD and early pediatric BPD and concluded that there may be different genetic mechanisms involved in early and later BPD onset [23].

As the emotional dysregulation of pediatric bipolar disorder is highly morbid, not intervening is usually not an option. In addition both early onset illness and delay to first treatment are independent risk factors for increased morbidity in adulthood, so properly diagnosing and treating children with BP in the early stages of illness is critical [24]. Yet treating children with BP disorder is challenging. Children with BP disorder are frequently treated with a variety of medications [25, 26]. As many as 50% of adolescents with mania require augmentation with more than one agent [25, 27]. Unfortunately, none of the current conventional treatments (commonly, anti-psychotics, lithium and anti-convulsants) can claim a high level of efficacy combined with easy tolerability in children. Mood stabilizers, such as lithium, have been the mainstay of pharmacotherapy for adults with BP disorder, but have showed only minimal effectiveness in children with BP disorder [28, 29]. Clinical trials of anticonvulsants and lithium in youth are marked by high drop-out, need for rescue medications and disappointing efficacy [30]. In a large retrospective Medicaid study of almost 7500 adolescents, patients started on valproate, oxcarbazepine and lithium frequently required augmentation with additional agents, had high discontinuation rates and had elevated risk of hospitalization relative to adolescents started on Second Generation Antipsychotics (SGAs) [28]. SGAs demonstrate significant reduction in manic symptoms and rapid onset of effect through the lifecycle [26, 28, 31]. While several newer atypical antipsychotic agents have received U.S. Food and Drug Administration (FDA) approval for the treatment of pediatric BP disorder, their use is also often fraught with significant and serious side effects including weight gain, dyslipidemia, glycemic dyscontrol, and risk for tardive dyskinesia [26].

Adverse effects and non-compliance in general are significant problems in the management of emotional regulation especially in pediatric populations. The side effects of weight gain, tremor, motor restlessness, acne, gastrointestinal distress and need for blood test monitoring can minimize adherence to treatment. Increasingly, clinicians, researchers and patients and their families are turning to an array of over the counter products considered complementary and alternative treatments. That dietary supplements appear to be safe and even healthful make them attractive options, especially for very young children, for those with only mild to moderate distress, or as supplements to conventional treatment. Yet, few studies exist to support their use in pediatric populations.

N-acetylcysteine (NAC) may be such an alternative. NAC is an acetylated amino acid and a precursor of glutathione. When ingested, NAC increases cysteine levels and allows for the synthesis of more glutathione in the brain. Glutathione, an antioxidant, acts to reduce oxidative stress, which has been implicated in BP disorder and major depression [32, 33]. Glutathione is poorly absorbed and rapidly metabolized when ingested, and thus is neither a viable nor helpful treatment option [34]. NAC, however, crosses the blood–brain barrier with ease and may be a more viable catalyst for this process [35].

NAC was FDA approved as a prescription drug in 1963 to treat acetaminophen overdose. For this reason, in July 2020, the Federal Drug Administration indicated that NAC cannot lawfully be marketed as a dietary supplement, however prior to this announcement, many different forms of oral NAC supplements were available over the counter and sold as a complementary and alternative treatment for a variety of medical conditions.

Clinical trials in adults have provided compelling evidence suggesting NAC’s efficacy as an evidence-based treatment for BP disorder. Open-label and double-blind, randomized, placebo-controlled trials of NAC have found decreases in depression rating scale scores and improvements in global functioning in adults with BP disorder and depressive symptoms [36, 37]. NAC’s safety in pediatric populations has also been demonstrated in trials of children and adolescents with autism spectrum disorders [38–40], obsessive–compulsive disorder (OCD) [41, 42], non-suicidal self-injurious behavior [43], and cannabis dependence [44]. These trials showed a positive response to treatment with NAC with excellent tolerability. NAC’s demonstrated helpfulness in treating a multitude of psychiatric disorders (OCD; marijuana, nicotine, and cocaine addictions; gambling; skin picking; nail biting; trichotillomania; schizophrenia; autism; and BP disorder) suggests that it may also be safe and effective in the treatment of pediatric BP disorder and worthy of further investigation [45]. Further adding to its appeal, NAC has evidence of positive impact on adverse events when used as a combination treatment with antipsychotics and possibly with lithium [32, 46].

The main aim of this study was to assess the safety, tolerability, and effectiveness of NAC in the treatment of pediatric BP spectrum disorder. To this end, we completed a 12-week, open-label trial in children and adolescents 5–17 years old with BP spectrum disorder. We hypothesized that NAC would be both a safe and effective treatment in this population and would be well tolerated. To the best of our knowledge, this is the first systematic assessment of NAC in the treatment of pediatric BP spectrum disorder.

## Methods

### Participants

Participants were male and female children and adolescents ages 5–17 meeting *Diagnostic and Statistical Manual of Mental Disorders, Fifth Edition* (*DSM-5*) diagnostic criteria for BP spectrum disorder (type I, type II, or not otherwise specified) and displaying mixed, manic, or hypomanic symptoms without psychotic features at the time of evaluation.

All bipolar spectrum disorder diagnoses were established by clinical interviews of the children and their parents or guardians by an expert clinician, supported by the mania and depression mood modules of the Kiddie Schedule of Affective Disorders and Schizophrenia, Epidemiological Version (K-SADS-E) [47, 48]. Participants were required to meet criteria for BP-I, BP-II or BP-NOS as defined by the DSM-5.

To ensure that participants with severe manic symptoms were not subjected to a trial with a treatment of unclear efficacy, eligible participants were required to have a Young Mania Rating Scale (YMRS) [49–51] total score ≥ 20 and ≤ 40 at baseline assessment. Participants with a score of 8 (“delusions; hallucinations”) on YMRS item 8 (content) were excluded from the study. All assessments were completed by board-certified or board-eligible child and adolescent psychiatrists trained to a high level of interrater reliability. The intraclass correlation score for interrater reliability on the YMRS was 0.81 [52].

Participants with any serious or unstable medical illness were excluded. Those with a history of sensitivity or intolerance to NAC, severe allergies, or multiple adverse drug reactions were also excluded from the study. Participants with an estimated full scale intelligence quotient (IQ) < 70 were excluded. Finally, active substance abusers, participants judged clinically to be at serious suicidal risk, and participants with a current diagnosis of schizophrenia were excluded from participation.

Concomitant psychotropic medications were allowed in this study as long as the participant’s treatment regimen remained the same throughout the entire study and had been stable for at least one month prior to study entry. Only participants with a poor response to their current medication treatment would be advised to consider a taper off their medications for entry into the study. However, no participants were tapered off their medications over the course of this study. Non-pharmacological treatments such as individual, family, or group therapy were allowed if they were in place before the participant joined the study. The participant’s therapy regimen was required to remain the same throughout the study. No new pharmacological or non-pharmacological treatments were to be initiated after study participation had begun. The use of the benzodiazepine lorazepam was permitted during the study at a maximum dosage of 2 mg per day for maximum of three days during the study. Any greater need for lorazepam was considered evidence of poor treatment response and grounds for termination from the study.

All study procedures were reviewed and approved by the committee for human subjects at our institution. All participants’ parents or guardians and participants ages 14 years or older signed written informed consent forms and participants ages 7 years to 13 years signed written assent forms. The trial was registered on ClinicalTrials.gov (Identifier: NCT02357290).

### Study design

All participants received open-label treatment with BioAdvantex brand N-acetylcysteine (“PharmaNAC”) in 900 mg effervescent tablets. Participants were instructed to dissolve each tablet in at least 8 oz of liquid (water, juice, or any carbonated drink). Participants ages 5–12 started with an initial dose of 900 mg daily for the first week of the study and were then titrated up to a maximum dose of 1800 mg daily in the second week of the study. Participants ages 13–17 followed the same titration schedule but were then titrated up to a maximum dose of 2700 mg daily in the third week of the study. These age-based maximum doses were maintained for the remainder of the trial and could be separated into two daily doses if preferred.

Study clinicians assessed safety and efficacy of the study treatment at weekly intervals. For the first four weeks of treatment, participants were seen in our office. After week 4, participants returned to the office monthly, and the study doctor conducted weekly visits over the phone with the subject’s parent or guardian between office visits. Participants were asked to return unused tablets at each office visit. Study medication was counted by study staff at every office visit to ensure compliance and participants who failed to keep study appointments or were non-compliant with treatment (less than 70% compliance for two weeks or longer) were discontinued from study. Adverse events and concomitant medications were monitored weekly.

### Clinician-rated assessment scales

Severity of manic and depressive symptoms were assessed weekly using the YMRS, the Massachusetts General Hospital Pediatric Mania Symptom Checklist (MSC), the Children's Depression Rating Scale (CDRS) [53, 54], and the Hamilton Depression Rating Scale (HDRS) [55]. Global functioning was assessed weekly using the Global Assessment of Functioning (GAF) [56]. ADHD and psychotic symptoms were evaluated at baseline, midpoint, and endpoint with the ADHD Rating Scale (ADHD-RS) [57] and the Brief Psychiatric Rating Scale (BPRS) [58], respectively. To determine clinically significant severity and improvement relative to baseline, the NIMH Clinical Global Impression (CGI) severity (CGI-S) and improvement (CGI-I) scales [59] were completed weekly. CGI-S and CGI-I were assessed separately for mania, depression, anxiety, ADHD, ODD, and overall BP spectrum disorder.

### Parent-rated scales

The parent or guardian of each participant completed the Pediatric Quality of Life Enjoyment and Satisfaction Questionnaire (PQ-LES-Q) [60] at baseline and endpoint to assess life enjoyment and satisfaction, the Behavior Rating Inventory of Executive Functioning – Parent Form (BRIEF-P) [61] at baseline and endpoint to assess executive functioning deficits, and the Social Responsiveness Scale, Second Edition – School-Age Form (SRS-2) [62] at screening and endpoint to assess capacities of social interaction. Socioeconomic status (SES) was measured at screening using the 5-point Hollingshead scale [63].

### Cognitive assessments

Full scale IQ was estimated at screening using the Kaufman Brief Intelligence Test, Second Edition (KBIT-2) [64] for participants age 5 and the Wechsler Abbreviated Scale of Intelligence, Second Edition (WASI-II) [65] for participants ages 6–17.

Cognitive functioning was assessed at screened and endpoint using subtests of the Wechsler Preschool and Primary Scale of Intelligence, Third Edition (WPPSI-III) [66] for participants age 5, subtests of the Wechsler Intelligence Scale for Children, Fourth Edition (WISC-IV) [67] for participants ages 6–16, and the Wechsler Adult Intelligence Scale, Fourth Edition (WAIS-IV) [68] for participants age 17. However, no participants age 5 and only one participant age 17 completed the cognitive functioning assessments at endpoint. Therefore, we only analyzed changes in cognitive functioning for participants ages 6–16 using the WISC-IV.

### Safety assessment

Safety was assessed at each visit using spontaneous reports of treatment-emergent adverse events. Changes in vital signs (blood pressure, temperature, height, and weight) were recorded at every office visit.

The Columbia-Suicide Severity Rating Scale (C-SSRS) [69] was administered weekly by a study clinician to assess initial and emergent suicidality in participants. Participants with scores for 4 or higher on the C-SSRS were discontinued from the study.

### Definition of clinical response

Response was defined as having either a ≥ 30% reduction in symptoms according to the YMRS, HDRS, or CDRS at endpoint or by a rating of “much improved” or “very much improved” (score ≤ 2) on the CGI-I for mania or depression.

Poor response to treatment was defined by a CGI-S score for overall BP spectrum disorder 2 points higher (more severe) than baseline for 2 weeks in a row or a YMRS score 30% higher than baseline for 2 weeks in a row, which was grounds for discontinuation from the study as determined by the study clinician. Participants with individual YMRS item scores of 8 on item 8 (content) or greater than 6 on item 9 (disruptive/aggressive behavior) for 2 weeks in a row were also discontinued from the study.

### Statistical analysis

Outcome measures and vital signs were analyzed using mixed-effects linear regression models with time as the predictor. For scales that were only collected at baseline and endpoint, participants were excluded from analysis if they were missing data at either time point. All models used robust standard errors to account for the repeated measures on each subject. Additionally, we performed a sensitivity analysis restricting the sample to participants receiving NAC monotherapy. Tests were two-tailed and performed at the 0.05 alpha level using Stata (Version 15.1) [70]. All analyses were intention-to-treat (ITT). Descriptive statistics are reported as absolute numbers, percentages, or mean ± standard deviation (SD) and use last observation carried forward (LOCF) for participants who did not complete the 12 weeks. Standardized mean differences (SMDs) were calculated as Cohen’s d comparing baseline and endpoint assessments; the calculation was the difference in mean values divided by the pooled standard deviation. In all cases, a positive SMD indicates an improvement from baseline to endpoint. As defined by Cohen [71], a SMD = 0.2 is interpreted as a small effect size, SMD = 0.5 as medium, and SMD = 0.8 as large.

## Results

Forty participants enrolled in the trial. Fourteen participants completed the 12-week study. Of the 26 participants who did not complete the study, six were deemed ineligible after enrollment, 14 withdrew from the study for various reasons, three were investigator-terminated, and three were lost to follow-up.

Of the 40 enrolled participants, nine received concomitant medications throughout their time in the study. Of the nine participants receiving concomitant medications, seven were only taking one concomitant medication (two participants were taking aripiprazole, two were taking ziprasidone and the others were taking buproprion, olanzapine and venlafaxine), one participant was taking two concomitant medications (aripiprazole and citalopram), and one was taking three concomitant medications (aripiprazole, buproprion and lamotrigine).

Analyses for this study included all participants who were exposed to treatment for two weeks or longer (*N* = 26). Of this group, five participants received concomitant medications throughout their time in the study, while the 21others received NAC monotherapy. None of the exposed participants withdrew or were terminated from the study due to adverse events (AEs). Of the final five included in the analyses, four were only taking one concomitant medication (two taking aripiprazole, one taking olanzapine, one taking ziprasidone,) and one was taking two concomitant medications (ariprazole and citalopram).

Of the 26 participants included in the analysis, 14 completed all 12 weeks, one completed 11 weeks, one completed six weeks, five completed four weeks, three completed three weeks, and two completed two weeks. The high drop-out rate suggests the need to design future trials which place lower burden on participants, with fewer study visits, fewer study measures and study visits accomplished via telepsychiatry.

### Baseline demographic and clinical characteristics

Participants were an average age of 10.0 ± 3.8 years and had an average SES of 1.9 ± 1.1. Twelve (46%) participants were male. Nineteen participants (73%) were Caucasian, three (11%) were Black or African American, one (4%) was American Indian or Alaskan Native, two (8%) reported being more than one race, and one (4%) did not report race. At baseline, participants had an average YMRS score of 23.8 ± 5.7, CDRS score of 37.1 ± 11.7, and HDRS score of 15.3 ± 7.9.

### Overall functioning

There were significant improvements in overall functioning as measured by the clinician-rated GAF (Baseline: 53.8 ± 5.8, Endpoint: 58.2 ± 8.3, Mean Difference: 4.3 ± 6.1; SMD [95% CI]: 0.61 [0.05, 1.16]; *p* < 0.001). Similarly, parents reported significant improvements in their children’s quality of life as measured by the PQ-LES-Q (*N* = 16; Baseline: 44.1 ± 7.0, Endpoint: 50.1 ± 7.9, Mean Difference: 6.0 ± 7.6; SMD [95% CI]: 0.81 [0.08, 1.52]; *p* = 0.002).

### Anti-manic and anti-depressant response to treatment

**Fig. 1:**
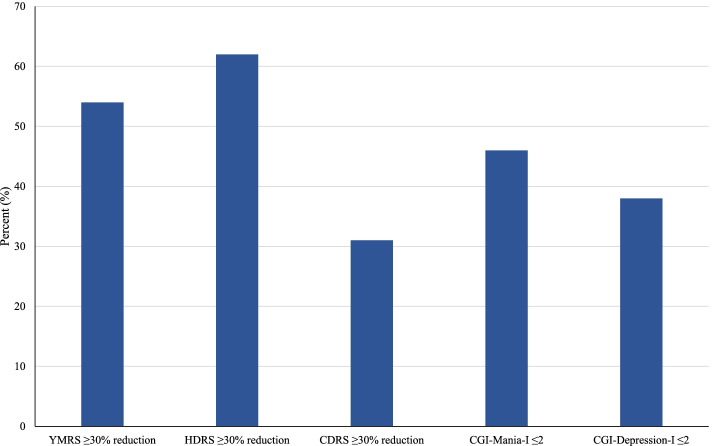
Bar graph depiction of YMRS, CDRS scores, reduction in HDRS scores, and CGI-I

As shown in Table 1, there were significant reductions in manic symptoms as measured by the YMRS and in depressive symptoms as measured by the CDRS and HDRS from baseline to endpoint. Furthermore, as shown in Fig. 1, 14 (54%) participants had a ≥ 30% reduction in scores on the YMRS, eight (31%) had a ≥ 30% reduction in CDRS scores, and 15 (62%) had a ≥ 30% reduction in HDRS scores. Upon examination of the CGI-I, 12 (46%) participants had CGI-Mania-I scores ≤ 2 and 10 (38%) had CGI-Depression-I scores ≤ 2 (Fig. 1). Improvement in manic symptoms was further supported by the clinician-rated MSC which showed a significant reduction in symptoms (Baseline: 17.1 ± 5.7, Endpoint: 11.8 ± 7.8, Mean Difference: -5.3 ± 6.6; SMD [95% CI]: 0.77 [0.21, 1.33]; *p* < 0.001).

**Table 1:** Change in YMRS, HDRS, and CDRS scores over time (*N* = 26)

	Baseline	Endpoint†	Difference	Cohen’s d (95% CI)	Test Statistic	*P*-Value
	Mean ± SD	Mean ± SD	Mean ± SD			
YMRS	23.8 ± 5.7	15.7 ± 10.1	-8.1 ± 8.5	0.99 (0.41, 1.56)	z = -7.19	< .001
HDRS	15.3 ± 7.9	9.5 ± 8.5	-5.8 ± 7.2	0.70 (0.14, 1.26)	z = -3.95	< .001
CDRS	37.1 ± 11.7	31.9 ± 14.7	-5.2 ± 10.7	0.39 (-0.16, 0.94)	z = -3.90	< .001

^†^ Endpoint mean uses last observation carried forward for subjects who dropped prior to week 12

### Response to treatment in other domains of psychopathology

**Fig. 2:**
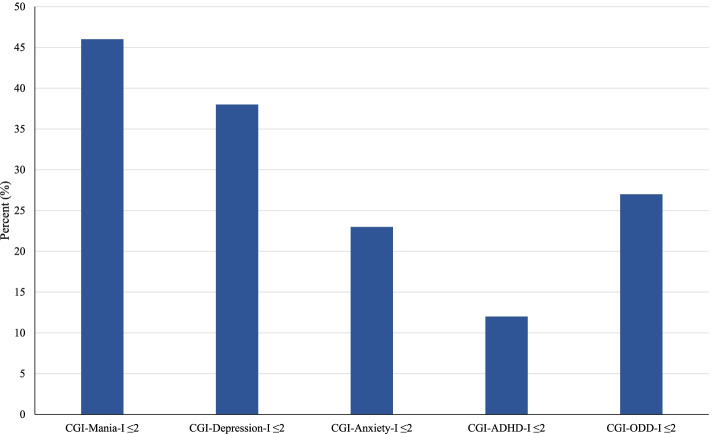
Bar graph depiction of percent of participants with CGI-I scores ≤ 2 for anxiety, ADHD, and ODD, ranging from 12% for ADHD to 27% for ODD

There were significant reductions in symptoms of overall psychopathology as measured by the clinician-rated BPRS (*N* = 19; Baseline: 43.4 ± 12.3, Endpoint: 34.8 ± 13.9, Mean Difference: -8.6 ± 10.8; SMD [95% CI]: 0.65 [-0.003, 1.30]; *p* < 0.001). Examining individual psychopathology, responses to treatment in domains other than mania and depression were less robust. Figure 2 shows the percent of participants with CGI-I scores ≤ 2 for anxiety, ADHD, and ODD, ranging from 12% for ADHD to 27% for ODD. While there were few participants with CGI-ADHD-I ≤ 2, there was a statistically significant improvement in ADHD symptoms on the clinician-rated ADHD-RS (*N* = 20; Baseline: 34.2 ± 15.0, Endpoint 27.2 ± 15.4, Mean Difference: -7.1 ± 14.0; SMD [95% CI]: 0.46 [-0.17, 1.09]; *p* = 0.03).

### Cognitive side effects

There were no cognitive side effects as demonstrated by the BRIEF-P, WISC-IV, and SRS. While there were statistically significant reductions in scores on seven of the eight BRIEF-P subscales and all three composite scales (*N* = 16; *p* < 0.05 on all composite and subscales except Self-Monitoring with *p* = 0.18), the reductions were small and the SMDs for these subscales were not clinically meaningful (0.21 to 0.69). There were no significant differences in scores from baseline to endpoint on the four WISC-IV executive functioning measures (*N* = 13; all *p* > 0.05) or the six SRS subscales (*N* = 18; all *p* ≥ 0.05). The SMDs for the WISC-IV measures ranged from -0.02 to 0.52, and the SMDs for the SRS subscales ranged from 0.03 to 0.28.

### Safety measures

AEs for participants who started the study medication for at least one week (*N* = 29) are reported in Table 2. Of those who started the study medication, 17 (59%) experienced at least one AE, reporting an average of 2.3 ± 1.6 AEs (range: 1–6). The most commonly reported AE was nausea/vomit/diarrhea.

**Table 2:** Adverse events in subjects who started study medication (≥ 1 week) (*N* = 29)

Adverse Event	Occurred Only 1 Time	Occurred ≥ 2 Times
Nausea/Vomit/Diarrhea (Gastrointestinal)	5 (17)	2 (7)
Insomnia	4 (14)	0 (0)
Cold/Infection/Allergy	3 (10)	1 (3)
Headache	3 (10)	0 (0)
Anxious/Worried	2 (7)	0 (0)
Neurological	2 (7)	0 (0)
Dizzy/Lightheaded	1 (3)	0 (0)
Musculoskeletal	1 (3)	0 (0)
Increased Appetite	0 (0)	1 (3)
Dermatological	0 (0)	1 (3)
Other: Thirsty	0 (0)	1 (3)
Other: Bike injury with need for stitches	1 (3)	0 (0)
Other: Dissociation	1 (3)	0 (0)

Over the course of the trial, there were no clinically or statistically significant changes in systolic blood pressure, diastolic blood pressure, pulse, or height (all *p* > 0.05). There was a statistically significant increase in weight (Baseline: 44.1 ± 25.9 kg, Endpoint: 45.0 ± 26.5 kg, Mean Difference: 0.9 ± 1.5 kg; SMD [95% CI]: -0.04 [-0.59, 0.52]; *p* = 0.01). While most participants saw small changes in weight, there were two who had outlying weight gains of 4.6 and 5.4 kg, one of whom was taking a concomitant medication. The participant with weight gain of 4.6 kg was also taking olanzapine (10 mg). The other participant with outlying weight gain was not taking any concomitant medications.

### Sensitivity analysis: NAC monotherapy

Results largely remained the same when we performed a sensitivity analysis restricting the sample to the 21 exposed participants who were receiving NAC monotherapy. Significance stayed the same for all outcome measures except for the BRIEF Self Monitor and Initiate subscales, which lost significance. Most of the effect sizes saw minimal changes, with the exception of the YMRS, BPRS and MSC, which dropped from 0.99, 0.65, and 0.77, respectively, in the whole group to 0.91, 0.54, and 0.69, respectively, in the group receiving NAC monotherapy. However, despite these decreases, the SMDs for the YMRS, BPRS, and MSC remained moderate to large in size.

## Discussion

Our results provide pilot data suggesting the safety and tolerability of NAC used in a pediatric population with mixed, manic or hypomanic states. In these pilot findings, with open-label conditions and small sample size, treatment with NAC led to significant improvements in our primary outcome measure of manic symptoms, but also depressive symptoms, and was very well tolerated. While preliminary from a pilot open label study, these findings suggest the need for larger randomized controlled trials. While this study addresses bipolar spectrum disorder with inclusion criteria focused on the presence of full and subthreshold manic symptomatology, the depression rating scales at baseline indicate significant mixed state depressive states, consistent with previous reports that pediatric bipolar disorder is most frequently mixed in presentation. Additionally, in this pilot study NAC treatment led to improvements in over functioning and improved quality of life. The results of this open-label, prospective clinical trial suggest that NAC is a well-tolerated potentially effective treatment for pediatric BP spectrum disorder. If confirmed in future controlled clinical trials, these results may provide an alternative treatment option in the management of mania and hypomania in youth with pediatric BP spectrum disorder, either as monotherapy or as an add-on treatment as demonstrated in this study.

**Fig. 3:**
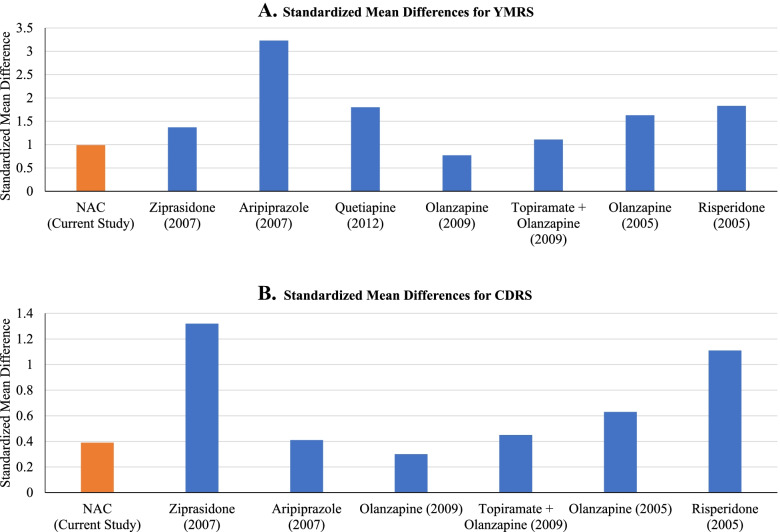
Two bar graphs depicting standardized mean differences in YMRS and CDRS scores in relation to antipsychotic medications and NAC

Although novel in a pediatric population, our findings are consistent with previous research in adult populations. NAC has been shown to improve depressive symptoms and global functioning in adults in open-label and double-blind, randomized, placebo-controlled trials [36, 37]. The current pilot findings suggest that NAC may also be effective for children and adolescents. An agent with minimal effect on the adult brain could play a major positive role in the developing brain, and future study can assess whether intervening with supplementation of safe complementary and alternative treatments such as NAC during critical periods may enhance brain development.

While effect sizes for mania and depression of atypical antipsychotic medications used for pediatric BP spectrum disorder in open label trials generally exceed those found with NAC, the SMDs for NAC for both the YMRS and CDRS are comparable to open-label trials of a subset of antipsychotic medications in pediatric BP disorder, and even exceed those from a trial of olanzapine, as illustrated in Fig. 3 [72–76]. This preliminary evidence of effectiveness indicates that NAC may prove to provide an important option in the treatment of youth with BP spectrum disorder, as an adjunctive treatment or in cases in which side effects make the use of mood stabilizing medications medically inadvisable. While complementary and alternative treatments may offer promising alternatives to, or additions to, prescription medications with low side effect liability, natural treatments with unclear and/or low efficacy should never discourage individuals from assessing the risks and benefits of treatment with FDA-approved known anti-manic agents with demonstrated efficacy.

Participants treated with NAC also improved symptoms of anxiety, ADHD, and ODD, which is noteworthy considering the high rates of these comorbidities in pediatric BP disorder [5, 8, 9] and the high levels of morbidity associated with these comorbid disorders [8].

NAC treatment in this pediatric population was well-tolerated, as indicated by a low frequency of AEs, no dropouts of exposed participants due to any AEs, and a lack of significant changes in vital signs, consistent with previous research on the use of NAC in children and adolescents with other psychiatric disorders [38–40, 42–44]. The only significant change in any measured vital signs was an increase in weight of an average of 0.9 kg over the course of a 12-week treatment period. To our knowledge, no other study of NAC has reported weight gain, and there is no clear pathophysiologic reason for weight gain, but an age matched controlled study of monotherapy NAC would be informative for this side effect.

In addition to potential efficacy for psychiatric symptoms and a benign safety profile, NAC may offer additional health benefits. In a report of NAC as an add-on to antipsychotic medication in schizophrenia, the authors concluded that NAC may act as a neuroprotective treatment for extrapyramidal symptoms [46]. Further, there is evidence from rat studies that NAC could play a role in attenuating lithium induced renal failure [77]. These results argue for the further study of NAC as an add-on treatment that could both ameliorate bipolar symptoms and moderate the side effects associated with standard mood stabilizer treatments.

The findings presented by the study should be considered in the context of methodological limitations. Participants received treatment in open-label fashion and there was no placebo control group. Our final sample consisted of children referred to our study and was largely Caucasian and thus our findings may not be generalizable to community samples. Our sample included both pre-pubertal and adolescent aged children, but given the small sample size, we cannot draw any conclusions regarding the relative efficacy of NAC in younger versus older children. Additionally, only those with mild to moderate YMRS scores (YMRS < 40) were allowed to participate, limiting the generalizability of our findings to youth with more severe symptoms. As we conducted only abbreviated structured interviews with a focus on diagnoses of pediatric BP spectrum disorder, we were unable to report thoroughly on comorbid conditions. Finally, our sample size was relatively small and was further limited by a high dropout rate, due to fluctuations in BP spectrum disorder with the need for more aggressive treatment as well as the ready availability of NAC over the counter. Given the safety associated with natural treatments, reducing obstacles to participation by utilizing telepsychiatry virtual visits and thus obviating the need for travel to academic medical centers could encourage more participants to join and stay in trials of natural treatments. Future research could also benefit by involving larger samples, both monotherapy and add-on trials, and continuing to follow participants who require more aggressive pharmacological treatment, but continue to augment with NAC treatment.

## Conclusions

Despite these limitations, the results of this study are encouraging and support the further scientific investigation of NAC as a treatment in youth with BP spectrum disorder. NAC was well-tolerated and the preliminary pilot findings of improvement in both manic and depressive symptoms lays the foundation for more robust study. If the results of this open label study are confirmed in future larger controlled, randomized placebo-controlled trials, NAC may offer a safe and effective alternative or augmenting treatment, for BP spectrum disorder in children and adolescents.

## Data Availability

The datasets used and/or analyzed during the current study are available from the corresponding author on reasonable request.

## Abbreviations

BP: Pediatric Bipolar
ADHD: Attention-deficit/hyperactivity Disorder
ODD: Oppositional Defiant Disorder
FDA: Food and Drug Administration
NAC: N-acetylcysteine
OCD: Obsessive-compulsive Disorder
DSM-5: Diagnostic and Statistical Manual of Mental Disorders, Fifth Edition
K-SADS-E: Kiddie Schedule of Affective Disorders and Schizophrenia, Epidemiological Version
YMRS: Young Mania Rating Scale
IQ: Intelligence Quotient
MSC: Mania Symptom Checklist
CDRS: Children's Depression Rating Scale
HDRS: Hamilton Depression Rating Scale
GAF: Global Assessment of Functioning
ADHD-RS: ADHD Rating Scale
BPRS: Brief Psychiatric Rating Scale
CGI: Clinical Global Impression scale
CGI-S: Clinical Global Impression – Severity scale
CGI-I: Clinical Global Impression – Improvement scale
PQ-LES-Q: Pediatric Quality of Life Enjoyment and Satisfaction Questionnaire
BRIEF-P: Behavior Rating Inventory of Executive Functioning – Parent Form
SRS-2: Social Responsiveness Scale, Second Edition – School-Age Form
SES: Socioeconomic Status
KBIT-2: Kaufman Brief Intelligence Test, Second Edition
WASI-II: Wechsler Abbreviated Scale of Intelligence, Second Edition
WPPSI-III: Wechsler Preschool and Primary Scale of Intelligence, Third Edition
WISC-IV: Wechsler Intelligence Scale for Children, Fourth Edition
WAIS-IV: Wechsler Adult Intelligence Scale, Fourth Edition
C-SSRS: Columbia-Suicide Severity Rating Scale
ITT: Intention-to-treat
SD: Standard Deviation
LOCF: Last Observation Carried Forward
SMD: Standardized Mean Difference
AE: Adverse Event
SGAs: Second Generation Antipsychotics

## References

[1] Biederman J, Birmaher B, Carlson GA, Chang KD, Fenton WS, Geller B, Hoagwood K, Hyman SE, Kendler KS, Koretz DS (2001). National Institute of Mental Health Research Roundtable on Prepubertal Bipolar Disorder. J Am Acad Child Adolesc Psychiatry.

[2] Lewinsohn P, Klein D, Seeley J (1995). Bipolar disorders in a community sample of older adolescents: Prevalence, phenomenology, comorbidity, and course. J Am Acad Child Adolesc Psychiatry.

[3] Merikangas KR, He JP, Burstein M, Swanson SA, Avenevoli S, Cui L, Benjet C, Georgiades K, Swendsen J (2010). Lifetime prevalence of mental disorders in U.S. adolescents: results from the National Comorbidity Survey Replication-Adolescent Supplement (NCS-A). J Am Acad Child Adolesc Psychiatry.

[4] Van Meter A, Moreira ALR, Youngstrom E. Updated Meta-Analysis of Epidemiologic Studies of Pediatric Bipolar Disorder. J Clin Psychiatry. 2019;80(3):18r12180. 10.4088/JCP.18r12180. 10.4088/JCP.18r12180 30946542

[5] Biederman J, Faraone S, Wozniak J, Mick E, Kwon A, Aleardi M (2004). Further evidence of unique developmental phenotypic correlates of pediatric bipolar disorder: Findings from a large sample of clinically referred preadolescent children assessed over the last 7 years. J Affect Disord.

[6] Dickstein DP, Rich BA, Binstock AB, Pradella AG, Towbin KE, Pine DS, Leibenluft E (2005). Comorbid anxiety in phenotypes of pediatric bipolar disorder. J Child Adolesc Psychopharmacol.

[7] Faedda GL, Baldessarini RJ, Glovinsky IP, Austin NB (2004). Pediatric bipolar disorder: phenomenology and course of illness. Bipolar Disord.

[8] Joshi G, Wilens T (2009). Comorbidity in pediatric bipolar disorder. Child Adolesc Psychiatr Clin N Am.

[9] Wozniak J, Biederman J, Kiely K, Ablon S, Faraone S, Mundy E, Mennin D (1995). Mania-like symptoms suggestive of childhood onset bipolar disorder in clinically referred children. J Am Acad Child Adolesc Psychiatry.

[10] Vaudreuil CAH, Faraone SV, Di Salvo M, Wozniak JR, Wolenski RA, Carrellas NW, Biederman J (2019). The morbidity of subthreshold pediatric bipolar disorder: A systematic literature review and meta-analysis. Bipolar Disord.

[11] Wozniak J, Uchida M, Faraone SV, Fitzgerald M, Vaudreuil C, Carrellas N, Davis J, Wolenski R, Biederman J (2017). Similar familial underpinnings for full and subsyndromal pediatric bipolar disorder: A familial risk analysis. Bipolar Disord.

[12] Brent DA, Perper JA, Goldstein CE, Kolko DJ, Allan MJ, Allman CJ, Zelenak JP (1988). Risk factors for adolescent suicide: A comparison of adolescent suicide victims with suicidal inpatients. Arch Gen Psychiatry.

[13] Brent D, Perper J, Moritz G, Allman C, Schweers J, Roth C, Balach L (1993). Psychiatric risk factors for adolescent suicide: a case-control study. J Am Acad Child Adolesc Psychiatry.

[14] Biederman J, Faraone SV, Chu MP, Wozniak J (1999). Further evidence of a bidirectional overlap between juvenile mania and conduct disorder in children. J Am Acad Child Adolesc Psychiatry.

[15] Biederman J, Mick E, Wozniak J, Monuteaux MC, Galdo M, Faraone SV (2003). Can a subtype of conduct disorder linked to bipolar disorder be identified? Integration of findings from the Massachusetts General Hospital Pediatric Psychopharmacology Research Program. Biol Psychiatry.

[16] Pliszka SR, Sherman JO, Barrow MV, Irick S (2000). Affective disorder in juvenile offenders: A preliminary study. Am J Psychiatry.

[17] Geller B, Tillman R, Bolhofner K, Zimerman B (2008). Child bipolar I disorder: prospective continuity with adult bipolar I disorder; characteristics of second and third episodes; predictors of 8-year outcome. Arch Gen Psychiatry.

[18] Birmaher B, Axelson D, Goldstein B, Strober M, Gill MK, Hunt J, Houck P, Ha W, Iyengar S, Kim E, Yen S, Hower H, Esposito-Smythers C, Goldstein T, Ryan N, Keller M. Four-year longitudinal course of children and adolescents with bipolar spectrum disorders: the Course and Outcome of Bipolar Youth (COBY) study. Am J Psychiatry. 2009;166(7):795–804. 10.1176/appi.ajp.2009.08101569. 10.1176/appi.ajp.2009.08101569 PMC2828047 19448190

[19] Wozniak J, Wolenski R, Fitzgerald M, Faraone SV, Joshi G, Uchida M, Biederman J. Further evidence of high level of persistence of pediatric bipolar-I disorder from childhood onto young adulthood: a five-year follow up. Scand J Child Adolesc Psychiatr Psychol. 2018;6(1):40–51. 10.21307/sjcapp-2018-005.PMID:33520750;PMCID:PMC7750699. 10.21307/sjcapp-2018-005 PMC7750699 33520750

[20] Wozniak J, Gonenc A, Biederman J, Moore C, Joshi G, Georgiopoulos A, Hammerness P, McKillop H, Lukas SE, Henin A (2012). A magnetic resonance spectroscopy study of the anterior cingulate cortex in youth with emotional dysregulation. Isr J Psychiatry Relat Sci.

[21] Uchida M, Hung Y, Green A, Kelberman C, Capella J, Gaillard SL, Gabrieli JDE, Biederman J (2021). Association between frontal cortico-limbic white-matter microstructure and risk for pediatric depression. Psychiatry Res Neuroimaging..

[22] Wozniak J, Faraone SV, Martelon M, McKillop HN, Biederman J (2012). Further evidence for robust familiality of pediatric bipolar I disorder: results from a very large controlled family study of pediatric bipolar I disorder and a meta-analysis [published correction appears in J Clin Psychiatry. 2015 Jul;76(7):e891-2]. J Clin Psychiatry.

[23] van Hulzen KJE, Scholz CJ, Franke B, Ripke S, Klein M, McQuillin A, Sonuga-Barke EJ, Kelsoe JR, Landén M, Andreassen OA, Lesch KP, Weber H, Faraone SV, Arias-Vasquez A, Reif A, PGC ADHD Working Group, PGC Bipolar Disorder Working Group (2017). Genetic Overlap Between Attention-Deficit/Hyperactivity Disorder and Bipolar Disorder: Evidence From Genome-wide Association Study Meta-analysis. Biol Psychiatry..

[24] Post RM, Altshuler LL, Kupka R (2021). 25 Years of the International Bipolar Collaborative Network (BCN). Int J Bipolar Disord.

[25] Kowatch RA, DelBello MP (2003). The use of mood stabilizers and atypical antipsychotics in children and adolescents with bipolar disorders. CNS Spectr.

[26] Liu HY, Potter MP, Woodworth KY, Yorks DM, Petty CR, Wozniak JR, Faraone SV, Biederman J (2011). Pharmacologic treatments for pediatric bipolar disorder: a review and meta-analysis. J Am Acad Child Adolesc Psychiatry.

[27] Kowatch RA, DelBello MP. Pharmacotherapy of children and adolescents with bipolar disorder. Psychiatr Clin North Am. 2005;28(2):385–97. 10.1016/j.psc.2005.02.001. 10.1016/j.psc.2005.02.001 15826738

[28] Chen H, Mehta S, Aparasu R, Patel A, Ochoa-Perez M (2014). Comparative effectiveness of monotherapy with mood stabilizers versus second generation (atypical) antipsychotics for the treatment of bipolar disorder in children and adolescents. Pharmacoepidemiol Drug Saf.

[29] Findling RL, Robb A, McNamara NK, Pavuluri MN, Kafantaris V, Scheffer R, Frazier JA, Rynn M, DelBello M, Kowatch RA (2015). Lithium in the Acute Treatment of Bipolar I Disorder: A Double-Blind. Placebo-Controlled Study Pediatrics.

[30] Kowatch RA, Suppes T, Carmody TJ, Bucci JP, Hume JH, Kromelis M, Emslie GJ, Weinberg WA, Rush AJ. Effect size of lithium, divalproex sodium and carbamazepine in children and adolescents with bipolar disorder. J Am Acad Child Adolesc Psychiatry. 2000;39(6):713–20. 10.1097/00004583-200006000-00009. 10.1097/00004583-200006000-00009 10846305

[31] Perlis RH, Welge JA, Vornik LA, Hirschfeld RM, Keck PE Jr. Atypical antipsychotics in the treatment of mania: a meta-analysis of randomized, placebo-controlled trials. J Clin Psychiatry. 2006;67(4):509–16. 10.4088/jcp.v67n0401. 10.4088/jcp.v67n0401 16669715

[32] Berk M, Kapczinski F, Andreazza AC, Dean OM, Giorlando F, Maes M, Yucel M, Gama CS, Dodd S, Dean B (2011). Pathways underlying neuroprogression in bipolar disorder: focus on inflammation, oxidative stress and neurotrophic factors. Neurosci Biobehav Rev.

[33] Smaga I, Pomierny B, Krzyzanowska W, Pomierny-Chamiolo L, Miszkiel J, Niedzielska E, Ogorka A, Filip M (2012). N-acetylcysteine possesses antidepressant-like activity through reduction of oxidative stress: behavioral and biochemical analyses in rats. Prog Neuropsychopharmacol Biol Psychiatry.

[34] Witschi A, Reddy S, Stofer B, Lauterburg BH (1992). The systemic availability of oral glutathione. Eur J Clin Pharmacol.

[35] Dean O, Giorlando F, Berk M (2011). N-acetylcysteine in psychiatry: current therapeutic evidence and potential mechanisms of action. J Psychiatry Neurosci.

[36] Berk M, Copolov DL, Dean O, Lu K, Jeavons S, Schapkaitz I, Anderson-Hunt M, Bush AI (2008). N-acetyl cysteine for depressive symptoms in bipolar disorder–a double-blind randomized placebo-controlled trial. Biol Psychiatry.

[37] Berk M, Dean O, Cotton SM, Gama CS, Kapczinski F, Fernandes BS, Kohlmann K, Jeavons S, Hewitt K, Allwang C (2011). The efficacy of N-acetylcysteine as an adjunctive treatment in bipolar depression: an open label trial. J Affect Disord.

[38] Ghanizadeh A, Moghimi-Sarani E (2013). A randomized double blind placebo controlled clinical trial of N-Acetylcysteine added to risperidone for treating autistic disorders. BMC Psychiatry.

[39] Hardan AY, Fung LK, Libove RA, Obukhanych TV, Nair S, Herzenberg LA, Frazier TW, Tirouvanziam R (2012). A randomized controlled pilot trial of oral N-acetylcysteine in children with autism. Biol Psychiatry.

[40] Nikoo M, Radnia H, Farokhnia M, Mohammadi MR, Akhondzadeh S (2015). N-acetylcysteine as an adjunctive therapy to risperidone for treatment of irritability in autism: a randomized, double-blind, placebo-controlled clinical trial of efficacy and safety. Clin Neuropharmacol.

[41] Ghanizadeh A, Mohammadi MR, Bahraini S, Keshavarzi Z, Firoozabadi A, Alavi Shoshtari A (2017). Efficacy of N-Acetylcysteine Augmentation on Obsessive Compulsive Disorder: A Multicenter Randomized Double Blind Placebo Controlled Clinical Trial. Iran J Psychiatry.

[42] Li F, Welling MC, Johnson JA, Coughlin C, Mulqueen J, Jakubovski E, Coury S, Landeros-Weisenberger A, Bloch MH (2020). N-Acetylcysteine for Pediatric Obsessive-Compulsive Disorder: A Small Pilot Study. J Child Adolesc Psychopharmacol.

[43] Cullen KR, Klimes-Dougan B, Westlund Schreiner M, Carstedt P, Marka N, Nelson K, Miller MJ, Reigstad K, Westervelt A, Gunlicks-Stoessel M (2018). N-Acetylcysteine for Nonsuicidal Self-Injurious Behavior in Adolescents: An Open-Label Pilot Study. J Child Adolesc Psychopharmacol.

[44] Gray KM, Carpenter MJ, Baker NL, Desantis SM, Kryway E, Hartwell KJ, McRae-Clark AL, Brady KT (2012). A double-blind randomized controlled trial of N-acetylcysteine in cannabis-dependent adolescents. Am J Psychiatry.

[45] Wozniak J, Yildiz A, Ruiz P, Nemeroff C (2015). Alternative and Complementary Treatments. Bipolar Book: History, Neurobiology, and Treatment.

[46] Berk M, Copolov D, Dean O, Lu K, Jeavons S, Schapkaitz I, Anderson-Hunt M, Judd F, Katz F, Katz P, Ording-Jespersen S, Little J, Conus P, Cuenod M, Do KQ, Bush AI. N-acetyl cysteine as a glutathione precursor for schizophrenia—a double-blind randomized placebo-controlled trial. Biol Psychiatry. 2008;64(5):361–8. 10.1016/j.biopsych.2008.03.004. 10.1016/j.biopsych.2008.03.004 18436195

[47] Orvaschel H, Puig-Antich J (1987). Schedule for Affective Disorders and Schizophrenia for School-Age Children: Epidemiologic Version.

[48] Orvaschel H (1994). Schedule for Affective Disorders and Schizophrenia for School-Age Children Epidemiologic Version.

[49] Gracious BL, Youngstrom EA, Findling RL, Calabrese JR (2002). Discriminative validity of a parent version of the Young Mania Rating Scale. J Am Acad Child Adolesc Psychiatry.

[50] Young RC, Biggs JT, Ziegler VE, Meyer DA (1978). A rating scale for mania: reliability, validity and sensitivity. Br J Psychiatry.

[51] Youngstrom EA, Danielson CK, Findling RL, Gracious BL, Calabrese JR (2002). Factor structure of the Young Mania Rating Scale for use with youths ages 5 to 17 years. J Clin Child Adolesc Psychol.

[52] Wozniak J, Faraone SV, Chan J, Tarko L, Hernandez M, Davis J, Woodworth KY, Biederman J (2015). A randomized clinical trial of high eicosapentaenoic acid omega-3 fatty acids and inositol as monotherapy and in combination in the treatment of pediatric bipolar spectrum disorders: a pilot study. J Clin Psychiatry.

[53] Emslie GJ, Rush AJ, Weinberg WA, Kowatch RA, Hughes CW, Carmody T, Rintelmann J (1997). A double-blind, randomized, placebo-controlled trial of fluoxetine in children and adolescents with depression. Arch Gen Psychiatry.

[54] Poznanski EO, Cook SC, Carroll BJ. A depression rating scale for children. Pediatrics. 1979;64(4):442–50. 492809

[55] Hamilton M (1960). A rating scale for depression. J Neurol Neurosurg Psychiatry.

[56] Endicott J, Spitzer RL, Fleiss JL, Cohen J (1976). The global assessment scale. A procedure for measuring overall severity of psychiatric disturbance. Arch Gen Psychiatry.

[57] DuPaul GJ, Power TJ, Anastopoulos A, Reed R (1998). The ADHD Rating Scale-IV Checklist, Norms and Clinical Interpretation.

[58] Lachar D, Bailley SE, Rhoades HM, Espadas A, Aponte M, Cowan KA, Gummattira P, Kopecky CR, Wassef A (2001). New subscales for an anchored version of the brief psychiatric rating scale: construction, reliability, and validity in acute psychiatric admissions. Psychol Assess.

[59] National Institute of Mental Health (1985). CGI (Clinical Global Impression) Scale - NIMH. Psychopharmacol Bull.

[60] Endicott J, Nee J, Yang R, Wohlberg C (2006). Pediatric Quality of Life Enjoyment and Satisfaction Questionnaire (PQ-LES-Q): reliability and validity. J Am Acad Child Adolesc Psychiatry.

[61] Roth RM, Isquith PK, Gioia GA (2004). Brief-A^tm^ Self report form. Psycholigcal Assessment Resources, Inc..

[62] Bruni TP (2014). Test Review: Social Responsiveness Scale-Second Edition (SRS-2). J Psychoeduc Assess.

[63] Hollingshead AB (1975). Four Factor Index of Social Status.

[64] Kaufman AS, Kaufman NL (2004). Kaufman Brief Intelligence Test, Second Edition (KBIT-2).

[65] Wechsler D (2011). Wechsler Abbreviated Scale of Intelligence - Second Edition (WASI-II).

[66] Wechsler D, Sattler JM (2002). WPPSI-III: Wechsler Preschool and Primary Scale of Intelligence.

[67] Wechsler D (2003). Wechsler Intelligence Scale for Children-Fourth Edition (WISC-IV).

[68] Wechsler D (2008). **Wechsler Adult Intelligence Scale - Fourth Edition (WAIS-IV)**, 4th.

[69] Posner K, Brent D, Lucas C, Gould M, Stanley B, Brown G, Fisher P, Zelazny J, Burke A, Oquendo M (2008). Columbia-Suicide Severity Rating Scale (C-SSRS).

[70] StataCorp (2017). Stata Statistical Software: Release 15.

[71] Cohen J (1988). **Statistical Power Analysis for the Behavioral Sciences**, Second.

[72] Biederman J, Mick E, Spencer T, Dougherty M, Aleardi M, Wozniak J (2007). A prospective open-label treatment trial of ziprasidone monotherapy in children and adolescents with bipolar disorder. Bipolar Disord.

[73] Biederman J, Mick E, Spencer T, Doyle R, Joshi G, Hammerness P, Kotarski M, Aleardi M, Wozniak J (2007). An open-label trial of aripiprazole monotherapy in children and adolescents with bipolar disorder. CNS Spectr.

[74] Joshi G, Petty C, Wozniak J, Faraone SV, Doyle R, Georgiopoulos A, Hammerness P, Walls S, Glaeser B, Brethel K (2012). A prospective open-label trial of quetiapine monotherapy in preschool and school age children with bipolar spectrum disorder. J Affect Disord.

[75] Wozniak J, Mick E, Waxmonsky J, Kotarski M, Hantsoo L, Biederman J (2009). Comparison of open-label, 8-week trials of olanzapine monotherapy and topiramate augmentation of olanzapine for the treatment of pediatric bipolar disorder. J Child Adolesc Psychopharmacol.

[76] Biederman J, Mick E, Hammerness P, Harpold T, Aleardi M, Dougherty M, Wozniak J (2005). Open-label, 8-week trial of olanzapine and risperidone for the treatment of bipolar disorder in preschool-age children. Biol Psychiatry.

[77] Efrati S, Averbukh M, Berman S, Feldman L, Dishy V, Kachko L, Weissgarten J, Golik A, Averbukh Z. N-Acetylcysteine ameliorates lithium-induced renal failure in rats. Nephrol Dial Transplant. 2005;20(1):65–70. 10.1093/ndt/gfh573. 10.1093/ndt/gfh573 15546888

